# Evaluation of the partnership between international non-governmental organizations and the State in the health sector in Mozambique

**DOI:** 10.11604/pamj.2021.39.91.25970

**Published:** 2021-06-01

**Authors:** Isabelle Munyangaju, Matias Alberto Seth Langa, Sue Ann Costa Clemens, Elisa Marchetti

**Affiliations:** 1 Tinpswalo Association, Vicentian Association to Fight AIDS and Tuberculosis, Gaza, Mozambique,; 2 NAIMA+, Network of INGOs Working in Health and HIV/AIDS, Maputo, Mozambique,; 3 Institute for Global Health, University of Siena, Siena, Italy,; 4 GlaxoSmithKline (GSK) Vaccines Institute for Global Health, Siena, Italy

**Keywords:** Mozambique, non-governmental organizations, health system strengthening

## Abstract

**Introduction:**

Mozambique is one of the poorest nations in the world and its health budget is heavily dependent on external funding. Increasingly, donors prefer to direct their funds through international non-governmental organizations instead of direct donations to the State budget. In the current climate of increased emphasis on health system strengthening, a strong and stable partnership between government and international non-governmental organizations is pivotal for health system strengthening in Mozambique.

**Methods:**

the study evaluates the current partnership through a standardized survey to healthcare workers employed by international non-governmental organizations in health (INGO, private) and the ministry of health (MOH, public). Results of the survey have been analyzed only descriptively and no statistical evaluations have been performed.

**Results:**

out of the valid 109 responses obtained 55.1% were from MOH cadres and 45.0% from INGO cadres. Most have been in the health sector for more than 5 years. Most of the respondents recognize that INGOs assist the government in strengthening the health system (71.6%), see the internal brain drain to INGOs and salary scale difference as major problems (70.6% and 78.0%); 87.2% reported that the coordination between INGOs and government needs to be improved. MOH cadres perceived the migration of cadres to INGOs and the need for improving coordination as major issues more acutely than their INGO counterparts (80.0% vs. 59.2% and 88.3% vs. 85.7% respectively). INGOs were perceived to offer better quality health services by 51.4% of respondents (of these 69.4% were INGO respondents). The quality of health services was alike between INGOs and MOH for 33% of the respondents.

**Conclusion:**

through the various efforts outlined the MOH and INGOs are moving towards an environment of mutual accountability, joint planning and coordination as well as harmonization of activities; but there are still challenges to be addressed. Prioritization and increased funding of the planning unit and planning and cooperation directorate as well as strategies for workforce retention are urgently needed.

## Introduction

Mozambique National Health System was created in 1975, after independence. Between 1977 and 1992 the country experienced a crippling civil war, needing significant aid to recover and reconstruct [1]. In 1987, the Mozambican government received its first International Monetary Fund (IMF) loan with conditions (to reduce government expenditure, salary caps on civil servants etc.). It was presumed that the international non-governmental organizations (INGOs) would fill the gaps created by the IMF´s promoted structural adjustment programs (SAPs); as these usually translate in severe cuts in a country´s health expenditure [1-5].

Mozambique faces one of the biggest health workforce crises in modern times. In 2010, the ratio of doctor to population was 3.95 per 100,000 inhabitants and ratio of nurses was 25 per 100,000. The IMF has been reluctant to lift the imposed ceilings on the wage bills as part of the economic reforms, if the government cannot show how it would sustain these in the long term [6,7]. The situation deteriorated when in 2016, aid to the country was suspended by the IMF and other major donors upon discovery of hidden debts [8].

The chronic human resource shortage is compounded by an internal brain drain from the public health sector to the private INGO sector. Leading to a loss in experienced cadres in managerial positions and crippling further the national health system [9-11].This study aims to describe the partnership between the national health system (public sector) and the INGOs in the health sector from the perspective of the healthcare workers.

## Methods

This is a cross sectional study undertaken between 1^st^ December 2015 and 1^st^ March 2016 by means of a standardized, semi-structured electronic questionnaire. The purpose of the study is to describe the current relationship between the ministry of health (MOH) and the INGOs in the health field. The questionnaire sought to address the following categories: 1) benefits of the partnership to the health system, 2) disadvantages of the partnership, 3) efficiency of the coordination of activities between MOH and INGOs, 4) impact of the wage disparity between MOH and INGOs, 5) differences of health programs between MOH and INGOs and 6) suggestions to improve the partnership.

The questionnaire was an anonymous nine-question web-based survey written in Portuguese and English. Demographic information was not collected to maintain anonymity of participants. Data on which entity (MOH or INGO) the healthcare worker was employed by as well as seniority in the health sector was collected. The study target population was healthcare workers in various positions in MOH (ministry of health, hospitals and health centres) and INGOs. The survey was distributed electronically by email listings and WhatsApp to healthcare workers employed by the MOH and INGOs in Maputo city (staff in the ministry, hospitals, health centres and INGO offices).

The anonymous data were charted for each question according to response, place of work, seniority in the post and years in the health sector. The completed electronic surveys were automatically saved and stored on the web-based survey database (SmartSurvey®). Data were exported into Excel® and analyzed quantitatively and descriptively to obtain aggregated results which described key findings.

## Results

A total of 255 healthcare workers were sent the questionnaire in the period between 1^st^ December 2015 and 1^st^ March 2016. Hundred and nine responses were collected and analyzed-giving a 42.8% response rate. Of the 109 respondents, 60 (55.1%) were MOH cadres and 49 (45.0%) were INGO cadres. Majority of the healthcare workers (64.2%) had been in the health sector for ≥5 years. About half (50.5%) had been in their post for ≥5 years and only a quarter (25.7%) had occupied their post for more than 10 years (Table 1).

**Table 1:** data on place of work and seniority

Variables	Number (N=109)	Percentage (%)
**Place of work**		
MOH	60	55.0
INGO	49	45.0
**Years of experience in the health sector**		
None	1	0.9
<5 years	38	34.9
5-10 years	31	28.4
>10 years	39	35.8
**Years of experience in current position**		
None	0	0.0
< 5 years	54	49.5
5-10 years	27	24.8
> 10 years	28	25.7

**Benefits of the partnership to the health system**: majority pointed out that INGOs contribute to the strengthening of the health system (70.0% are MOH respondents and 73.5% are INGO respondents), offer technical and financial support to the Ministry of Health (66.0% are MOH respondents and 65.3% are INGO respondents), offer quality healthcare (58.7%: 71.4% are INGO respondents and 48.3% are MOH respondents), facilitate healthcare access in remote areas (56.0%: 67.3% are INGO respondents and 46.7% are MOH respondents) and contribute to training/capacity building (50.5%: 63.3% are INGO respondents and 40.0% are MOH respondents). Other benefits scored below 50.0% (Table 2).

**Table 2:** data on the characteristics of the partnership evaluated

Variables	Number (N=109)	Percentage (%)
**Advantage of INGOs for the health system in Mozambique**		
None	0	0.0
Strengthen the health system	78	71.6
Financial and technical support	72	66.1
Provision of quality health care	64	58.7
Training to healthcare workers	55	50.5
Research and innovation	47	43.1
Patient advocacy	36	33.0
Access to new treatments	53	48.6
Access to healthcare in remote areas	61	56.0
Other	7	6.4
**Disadvantages of INGOs for the health system in Mozambique**		
None	3	2.8
Fragmentation of the health system	19	17.4
Increase in parallel (vertical) programs	38	34.9
Migration of government health workers to INGOs	77	70.6
Increased workload on the local health care workforce	21	19.3
Lack of harmony with the ministry´s objectives and plan of action	32	29.4
Accountability to donors but less so to the ministry	27	24.8
Lack of monitoring of INGO activities by the ministry	31	28.4
Ministry has no influence over INGO funds and how they are utilized	26	23.9
Short-term contracts for INGO staff	43	39.4
INGOs have short term goals	38	34.9
INGOs need for instant results	39	35.8
Other	10	9.2
**Coordination of activities and objectives between MOH and INGOs**		
Very effective	6	5.5
Effective but need improvement	95	87.2
Poor or not effective	8	7.3
**Any obstacles brought about by the wage difference between MOH and INGOs**		
None	24	22.0
Limited obstacles	85	78.0
**Health programs run by MOH vs. INGOs**		
They are comparable in terms of quality and efficiency	36	33.0
They are not comparable in terms of quality and efficiency and I would give preference to programs run by the ministry of health	17	15.6
They are not comparable in terms of quality and efficiency and I would give preference to programs run by INGO	56	51.4
**Suggestions to make partnership more beneficial**		
Respondents	109	100.0

**Disadvantages of the partnership**: majority of respondents (70.6%) saw internal migration of MOH workers to the INGOs as a major drawback. This is the biggest concern among the MOH respondents (80.0%) but also among the INGO respondents (59.2%). Other disadvantages scored below 50.0% (Table 2).

**Efficiency of the coordination of activities between MOH and INGOs**: the bulk of respondents [95 (87.2%)] recognized that the coordination was effective but still needed improvement; of these: 88.3% were MOH respondents and 85.7% were INGO respondents. Coordination of activities means ensuring that there is no duplication of activities between MOH and its partners by joint planning, joint supervisions and joint evaluations of health strategies and programs.

**Impact of the wage disparity between MOH and INGOs**: majority [85 (78.0%)] saw the difference in salary scales as a problem, of these: 44 (83.7%) were INGO respondents and 41 (73.3%) were MOH respondents. Of those who reported the wage disparity as a problem, 103 (67.3%) reported that it led to MOH human resources demotivation, migration to the INGOs for higher pay and was a cause of conflicts between INGO and MOH healthcare workers (Table 2).

**Differences of health programs between MOH and INGOs**: more than half of the respondents [56 (51.4%)] felt that the INGOs offered better health services than the MOH and of these: 69.4% were INGO respondents and 36.7% were MOH respondents. However, 36 (33.0%) felt that the services were comparable i.e. one was not better than the other and majority of these (26 respondents) were MOH respondents. Seventeen (15.6%) felt that the MOH services were of better quality, and majority of these 12 were MOH respondents. The most reported reason [50 (52.2%)] for the difference in service quality is that INGOs have more resources to offer better services (Table 2).

**Suggestions to improve the partnership**: there was 100.0% response rate to this question. The responses were grouped as follows, in order of importance: 1) the MOH should have more ownership (demand more accountability and transparency with funds and projects, re-evaluate the benefits of INGOs, review the salary scales, and decrease bureaucracy)(-80 (41.2%)); 2) both INGOs and MOH should integrate and harmonize more their activities, do joint coordination, exchange information, share experiences, increase joint field presence and be more patient centered (-69 (35.6%)); 3) the INGOs should contribute more to health system strengthening, capacity building and have long-term objectives and contracts (-30 (15.5%)); 4) no comments (-15 (7.7%)).

## Discussion

There is an acknowledgement from both INGO and MOH health personnel, of the benefits that INGOs bring to the national health system of Mozambique in various areas. However, the survey clearly showed that there was poor dissemination of what INGOs do as reflected by the comparison of response rate between MOH vs. INGO within the same questions. This may well be that MOH workers may not be aware of such initiatives from the INGOs because they are not widely communicated. Conversely, there may be difference in perception of what the INGOs think they are doing and what the recipients or the MOH understand the INGOs to be doing. An additional reason may be because the survey was conducted in Maputo, where most INGOs have their coordination offices but not necessarily their field projects (which may be located in areas outside Maputo).

In the last decade, changes in policies and strategies both locally and internationally have brought about significant improvements to the partnership. These are for example: a) between 2008 and 2011 there were indications that donor and governmental resources were converging towards areas of most need, partly due to greater coordination between MOH and donors [12]. b) The various codes of conducts that have been developed [4,13,14]. c) Donors and multilateral agencies are increasingly allocating funds towards health system strengthening [4,15]. d) Integration of parallel programs such as HIV/AIDS into the mainstream health system (horizontal approach) allowing funds allocated to HIV/AIDS to benefit the national health system at large. This approach is supported by most actors in recent years including Global Fund, GAVI Alliance, World Bank, WHO and the government of Mozambique [13]. e) Government´s commitment to integration and decentralization of health services closer to the people i.e. from central level to provincial and district levels [16]. f) The Mozambique Ministry of Health has a system of gathering information on donor funds, which it uses to track the use of funds and for future planning of activities. The Ministry of Economy and Finance (formerly Ministry of Planning and Development) also has a similar tracking system which is an online tool. These are clear attempts to gather information on available financial resources and how they are used; as well as using this information to plan for the future [17].

The coordination of activities between MOH and INGOs is taking place but there is place for improvements. MOH and INGOs do joint planning through the various technical working groups and they carry out joint supervision and technical support visits in the health facilities. These jointly coordinated activities ensure alignment of strategies and avoid unnecessary duplication of activities. There is still a need to increase the frequency and consistency of the supervision visits at central and provincial levels. The MOH and INGOs would also benefit significantly from joint evaluations of the programs and strategies implemented, in order to strengthen the health system further by scaling up successful strategies and discontinuing those that were ineffective. To address the human resource shortage and brain drain, the government of Mozambique has instituted some corrective measures such as increasing the number of training institutions for doctors and nurses, training of medical assistances referred to as “Tecnicos de Medicina” (a cadre that takes a shorter duration to train than a doctor would take but can exercise most of the basic activities of a doctor), incentives for managerial positions and limiting the hiring of MOH staff by INGOs as per the various codes of conduct (e.g. Kaya Kwanga code of conduct) [11,16]. Despite all these efforts this remains a significant problem and there is still room for improvement. The government hiring mechanisms are still marred by bureaucracy and time delays. Donors do allocate funds towards capacity building but it is disease specific and doesn´t include workforce expansion. As a result of short term projects and a need for instant outputs, INGOs have taken to task shifting as well but in most cases it is done without taking into consideration the national health system´s capacity to absorb these new cadres and sustain them in the long run [18-20].

In terms of health planning, the MOH possesses a Planning Unit and therefore recognizes the need for a specialized unit with specialized skills. The strategic plans are drawn up in consultation with donors and INGOs, so that activities are harmonized and there is no duplication of actions. The lack of funds means that the process is also heavily reliant on donors. The Planning Unit has limited financial and human resource capacity, and thus relies on donors to provide fees for the hiring of consultants or to provide skilled staff to aid the planning and monitoring process. An additional challenge is that the strategic planning process does not involve all the stakeholders especially the community-it is mainly a central level exercise. However, with decentralization taking place it will have to involve central, provincial and district levels. This will require more finances and more skilled human resources (therefore more investment into capacity building) [21]. According to one study by Asad and Kay the Mozambique government would best be classified as a “willing and incapable” State. The structures and systems are there but need capacitation, technical and financial support to function effectively; and this is where the INGOs (and the donors) can make a significant impact. This can be addressed through joint coordination, joint supervision and program monitoring, exchange of skills and experiences [22].

In the last two years (2019 and 2020), this partnership has been put to test by a series of humanitarian crises that hit Mozambique - namely two back to back strong tropical cyclones in 2019 (Idai and Kenneth) and the COVID-19 pandemic in 2020. The crises have highlighted the strength of the partnership in coordinating and mounting an urgent response as well as exploiting years of investment in programs such as HIV/AIDS, Tuberculosis and Malaria. The COVID-19 response relied heavily on existing HIV/AIDS, Tuberculosis, Malaria and Cholera program structures to quickly make significant progress. Treatment centres, testing capacity and epidemiological vigilance were scaled up at an unprecedented speed and have allowed the country to limit the devastation that COVID-19 would have brought on the back of the cyclones [23-26]. The study had limitations. The small study sample was due to limited data collection time and the fact that it was conducted in Maputo City for ease of internet access. The survey did not identify participants by positions e.g. managerial to see if this had any bearing on the results. Statistical group analysis to know if there were major differences in responses between the two groups would have given more power to this study. A future survey could be designed taking this into consideration. Participants were from Maputo City which is the capital city of Mozambique and better resourced; it is not representative of the views of the rest of the country. Future studies should seek to replicate the findings in a larger study that includes various provinces as well as stratify the respondents by professional work functions. Additionally, it is important that future research evaluates the impact of the presence of the INGOs to the Mozambican health system after more than 30 years including a cost-benefit analysis. Such evaluations are an important step towards a deeper assessment of the current NGO-MOH partnership model in the 21^st^ century and promote reforms if necessary.

## Conclusion

Through the various efforts outlined the MOH and INGOs are moving towards an environment of mutual accountability, joint planning and coordination as well as harmonization of activities; but there are still challenges to be addressed. Prioritization and increased funding of the planning unit and planning and cooperation directorate as well as strategies for workforce retention are urgently needed. Joint program evaluations would further strengthen not only the partnership but also the health system at large.

### What is known about this topic

- The severe human resource shortage is one of the challenges crippling the health system in Mozambique;
- The internal brain drain from the public health sector to the private sector.

### What this study adds

- The internal brain drain from the public health sector to private sector is still a significant problem and needs to be addressed by health system reforms particularly in HR recruitment, management and retention;
- The MOH-NGO partnership is important and needs regular joint reviews to ensure continued benefits for all stakeholders;
- There is a need for both parties to engage in a deeper analysis of the partnership in the last 30 years in terms to assess whether the current model is still applicable and how it can be optimized. It should be assessed whether the current model can adequately respond to emerging epidemic threats and natural disasters resulting from climate change.
